# A prognostic model combining CD4/CD8 ratio and N stage predicts the risk of distant metastasis for patients with nasopharyngeal carcinoma treated by intensity modulated radiotherapy

**DOI:** 10.18632/oncotarget.9695

**Published:** 2016-05-30

**Authors:** Chang-Juan Tao, Yuan-Yuan Chen, Feng Jiang, Xing-Lai Feng, Qi-Feng Jin, Ting Jin, Yong-Feng Piao, Xiao-Zhong Chen

**Affiliations:** ^1^ Department of Radiation Oncology, Zhejiang Cancer Hospital, Key Laboratory of Radiation Oncology of Zhejiang Province, Hangzhou, Zhejiang Province, People's Republic of China

**Keywords:** nasopharyngeal carcinoma, lymphocyte subset, CD4/CD8 ratio, distant metastasis, survival

## Abstract

This study aimed to evaluate the correlation between circulating lymphocyte subsets and clinical variables, and design an effective prognostic model for distant metastasis-free survival (DMFS) in NPC. In this study, subsets of circulating lymphocytes were determined in 719 non-metastatic NPC patients before treatment. Overall survival and DMFS was monitored. Significant prognostic factors were identified using univariate and multivariate analyses. Results showed that the percentage of CD19^+^ lymphocytes correlated negatively with TNM stage (r = −0.082, *P* = 0.028). Patients with higher CD4/CD8 ratios (≥ 1.77) showed better 5-year DMFS than patients with lower ratios (91.9% *vs.* 85.4%, *P* < 0.001). Multivariate analysis revealed that CD4/CD8 ratio (HR, 0.450; 95% confidence interval [CI], 0.266–0.760; *P* = 0.003) and N classification (HR, 2.294; 95% CI, 1.370–3.839; *P* = 0.002) were independently prognostic factors for DMFS. The prognostic N-R model was developed and divided patients into three groups: (1) low-risk (early N stage and CD4/CD8 ratio ≥ 1.77); (2) intermediate-risk (advanced N stage or CD4/CD8 ratio < 1.77) and (3) high-risk (advanced N stage and CD4/CD8 ratio < 1.77) of distant metastasis. In conclusion our prognostic model, based on clinical N stage and CD4/CD8 ratio, may predict the risk of distant metastasis, allowing individualized treatment for NPC.

## INTRODUCTION

Nasopharyngeal carcinoma (NPC) is a distinct type of head and neck cancer owing to its extremely unbalanced endemic distribution, pathology and clinical attributes [1]. It has a high incidence rate, ranging from 20 to 30 cases per 100 000 people in southern China and the surrounding areas [1, 2]. Radiotherapy is the standard treatment modality for early NPC, and combined radiotherapy and chemotherapy is applied for loco-regional advanced disease [3, 4].

Given the current availability of conformal intensity-modulated radiotherapy, which accurately delivers high doses to the tumor target volume and spare normal tissue, distant metastasis has become the major cause of failure of treatment for NPC. More than 20% local-advanced NPC patients will develop distant metastasis after definitive chemoradiotherapy [4–6]. The tumor, lymph node and metastases status display prognostic relevance in NPC. However, within the same TNM stage there is heterogeneity in the duration of overall survival and risk of distant metastasis among patients with NPC [7]. Therefore, there is much interest in developing additional prognostic factors to permit more accurate patient stratification to evaluate individual prognosis. Such factors will improve clinical decision-making, and may help guide the provision of individualized treatment [8].

Immune cells, particularly T and B lymphocytes, play an important role in the immunological surveillance and can help in the elimination of tumor cells [9–11]. Previous studies have revealed that higher levels of particular lymphocyte subpopulations in the circulating blood were associated with tumor development and poor prognosis in NPC patients [10, 12]. However, these studies were only performed in a small number of patients (n=356 and n=94, respectively) [10, 12]. Despite these associations, the influence of lymphocyte subpopulations on metastatic prediction in patients with NPC remains unclear.

The relationship between lymphocyte subpopulations and NPC prognosis merits further exploration. In this study, we aimed to examine the correlation between lymphocyte subset distribution and clinical variables in order to design a convenient and effective prognostic model predicting distant metastasis in NPC patients.

## RESULTS

Patients' clinical characteristics are shown in Table 1. The median duration of follow-up for the entire patient group was 48 months (ranging from 3 to 89 months). A total of 81 (11.3%) patients died. Seventy-six (10.6%) patients developed local-regional failure, 60 (8.3%) patients developed distant metastases and 14 (1.9%) patients developed both local and distant failures. The median DMFS of the whole cohort was 45.0 months, ranging from 3.0 months to 88.0 months. The interquartiles (P25-P75) of DMFS was 34.0 months and 56.0 months respectively. For the entire cohort, the 5-year disease-free survival (DFS), overall survival (OS), local recurrence-free survival (LRFS) and DMFS rates were 77.6%, 85.8%, 86.7% and 89.4%, respectively.

**Table 1:** Baseline characteristics of patients (N = 719)

Characteristic	No. of patients
Age (≤50/>50 years)	382/337
Sex (male/female)	495/224
Mean percentage of NK cell (SD)	24.8 (10.5)
Mean percentage of CD3 (SD)	65.2 (10.4)
Mean percentage of CD4 (SD)	36.0 (8.1)
Mean percentage of CD8 (SD)	19.1 (7.8)
Mean percentage of CD19 (SD)	8.5 (3.9)
Mean percentage of CD25 (SD)	22.7 (7.1)
Mean percentage of CD44 (SD)	73.3 (12.0)
T stage* (T1/2/3/4)	73/107/353/186
N stage* (N0/1/2/3)	80/368/213/58
Clinical TNM stage* (I/II/III/IVa-b)	14/98/381/226

SD, standard deviation.

* According to the 7th AJCC/International Union against Cancer staging system.

The correlations between the percentages of circulating CD3^+^ T cells, CD4^+^ T cells, CD8^+^ T cells, CD19^+^ B cells, CD25^+^ T cells, CD44^+^ T cells, natural killer (NK) cell and CD4/CD8 ratio and clinical parameters are shown in Table 2. The percentages of CD3^+^ T cells and CD4^+^ T cells correlated negatively with clinical T stage (*r* = −0.090, *P* = 0.016; *r* = −0.082, *P* = 0.028, respectively), while the percentage of NK cells correlated positively with clinical T stage (*r* = 0.113, *P* = 0.002). The percentages of NK cells and CD4/CD8 ratio correlated negatively with clinical N stage(*r* = −0.075, *P* = 0.044; *r* = −0.013, *P* = 0.005, respectively). Contrarily, the percentages of CD8^+^ T cells and CD44^+^ T cells correlated positively with clinical N stage (r = 0.095, *P* = 0.011; *r* = 0.080, *P* = 0.033, respectively). The percentages of CD19^+^ lymphocytes correlated negatively with TNM stage (r = −0.082, *P* = 0.028).

**Table 2:** Correlation of immune cell subpopulations with clinical parameters

Clinical parameters	Immune cell subpopulations							
	CD3	CD4	CD8	CD19	CD25	CD44	NK	CD4/CD8 ratio
Sex	***r* = 0.156**	***r* = 0.109**	*r* = 0.044	*r* = 0.068	*r* = −0.017	***r* = 0.141**	***r* = −0.150**	*r* < 0.001
	***p* < 0.001**	***p* = 0.003**	*p* = 0.239	*p* = 0.067	*p* = 0.655	***p* < 0.001**	***p* < 0.001**	*p* = 0.994
Age	***r* = −0.095**	*r* = −0.025	*r* = −0.046	*r* = 0.026	***r* = 0.137**	*r* = −0.057	*r* = 0.044	*r* = 0.020
	*p* = 0.010*	*p* = 0.500	*p* = 0.220	*p* = 0.487	***p* < 0.001**	*p* = 0.129	*p* = 0.240	*p* = 0.592
Clinical T stage*	***r* = −0.090**	***r* = −0.082**	*r* = −0.038	*r* = −0.030	*r* = −0.045	*r* = −0.069	***r* = 0.113**	*r* = −0.012
	***p* = 0.016**	***p* = 0.028**	*p* = 0.308	*p* = 0.420	*p* = 0.224	*p* = 0.063	***p* = 0.002**	*p* = 0.742
Clinical N stage*	*r* = 0.053	*r* = −0.054	***r* = 0.095**	*r* = −0.052	*r* = −0.014	***r* = 0.080**	***r* = −0.075**	***r* = −0.103**
	*p* = 0.156	*p* = 0.148	***p* = 0.011**	*p* = 0.163	*p* = 0.715	***p* = 0.033**	***p* = 0.044**	***p* = 0.005**
TNM stage*	*r* = −0.040	*r* = −0.068	*r* = −0.004	***r* = −0.082**	*r* = −0.028	*r* = −0.057	*r* = 0.072	*r* = −0.041
	*p* = 0.288	*p* = 0.068	*p* = 0.906	***p = 0.028***	*p* = 0.448	*p* = 0.130	*p* = 0.055	*p* = 0.268

* According to the 7th AJCC/International Union against Cancer staging system.

**Figure 1:**
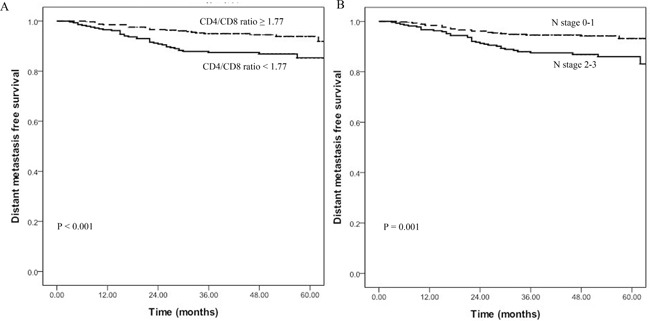
**A.** Correlation between distant metastasis-free survival (DMFS) for patients and CD4/CD8 ratio showing that patients with a higher CD4/CD8 ratio (≥ 1.77) have a better 5-year DMFS compared to those with a lower ratio (91.9% vs. 85.4%, *P* < 0.001). **B.** DMFS for patients with early N stage vs. advanced N stage showing that patients with advanced N stage (N2-3) display poorer 5-year DMFS compared with patients with early N stage 0-1 (93.2% vs. 83.1%, *P* = 0.001).

The cutoff points of circulating immune subsets (percentages of circulating CD3^+^ T cells, CD4^+^ T cells, CD8^+^ T cells, CD19^+^ lymphocytes, CD25^+^ T cells, CD44^+^ T cells, NK cells and CD4/CD8 ratio) were dichotomised (based on the ROC analysis) as shown in Table 3. Univariate analysis suggested that the percentage of circulating CD4^+^ T cells (*P* < 0.001), the percentage of circulating NK cells (*P* = 0.050), the CD4/CD8 ratio (*P* < 0.001) and clinical N classification (*P* = 0.001) were significantly associated with DMFS (Table 3). The clinical T classification showed a trend for association with DMFS (*P* = 0.052). The optimal cut-off value of CD4/CD8 ratio based on the ROC analysis was 1.77, with sensitivity of 60.8% and specificity of 61.7%. Patients with a higher CD4/CD8 ratio (ratio ≥ 1.77) showed better 5-year DMFS compared with patients with a lower CD4/CD8 ratio (91.9% vs. 85.4%, *P* < 0.001) (Figure 1A). When the best optimal cutoff was increased (CD4/CD8 ratio = 1.86 with the sensibility of 56.1% and specificity of 65.0%) or decreased (CD4/CD8 = 1.68 with the sensibility of 64.8% and specificity of 53.3%) by 5%, patients wiht higher CD4/CD8 ratio still had better 5-year DMFS compared with patients with lower CD4/CD8 ratio. The 5-year DMFS of patients with CD4/CD8 ratio ≥ 1.68 was higher than those with CD4/CD8 ratio < 1.68 (90.5% vs. 87.3%, *P* = 0.003). The results was similar when the cut off value was 1.86 (5-year DMFS: 91.9% vs. 86.3%; *P* = 0.001). Patients with more advanced N stage (N2-3) displayed poorer 5-year DMFS compared with patients with clinical N stage 0-1 (93.2% vs. 83.1%, *P* = 0.001) (Figure 1B).

**Table 3:** Univariate and multivariate analysis of factors influencing distant metastasis-free survival (DMFS)

Factors	No.	Univariate		Multivariate		
		5-year DMFS	*P*	HR	95% CI	*P*
Sex						
Male	495	87.3%	0.058			
Female	224	91.5%				
KPS						
≥ 90	694	90.3%	0.523			
< 90	25	87.2%				
Age (years)						
< 50	382	89.7%	0.645			
≥ 50	337	90.1%				
Therapy						
CCRT	698	90.4%	0.704			
RT alone	21	83.8%				
Induced CT						
Yes	649	90.0%	0.386			
No	70	93.9%				
Adjuvant CT						
Yes	183	86.4%	0.307			
No	536	91.2%				
LDH (IU/L)						
< 187	500	89.8%	0.271			
≥ 187	218	92.0%				
CD4^+^ T cells (%)						
< 32.5	266	82.3%	< 0.001			
≥ 32.5	453	92.7%				
CD8^+^ T cells (%)						
< 19.5	411	91.5%	0.098			
≥ 19.5	308	88.8%				
CD44^+^ T cells (%)						
< 65.5	158	93.3%	0.187			
≥ 65.5	561	89.7%				
NK cell (%)						
< 12.5	85	85.4%	0.050	0.554	0.293 - 1.049	0.070
≥ 12.5	634	89.6%				
CD4/CD8 ratio						
< 1.77	295	85.4%	< 0.001	0.450	0.266 - 0.760	0.003
≥ 1.77	424	91.9%				
T stage*						
T 1-2	180	91.7%	0.052			
T 3-4	539	88.7%				
N stage*						
N 0-1	446	93.2%	0.001	2.294	1.370 - 3.839	0.002
N 2-3	273	83.1%				

KPS, Karnofsky performance scale; LDH, lactic dehydrogenase; CI, confidence interval; HR, hazard ration; SD, standard deviation; CCRT, Concurrent Chemoradiotherapy; RT: radiotherapy; CT, chemotherapy.

* According to the 7th AJCC/International Union against Cancer staging system.

To identify independent metastatic prognostic factors, the variables that were found to be significant on univariate analysis were subjected to multivariate analysis. Because there is a duplication between the CD4^+^ lymphocytes and CD4/CD8 ratio, only CD4/CD8 ratio was entered into the multivariate analysis. Multivariate analysis revealed that CD4/CD8 ratio (HR, 0.450; 95% confidence interval [CI], 0.266–0.760; *P* = 0.003) and N stage (HR, 2.294; 95% CI, 1.370 – 3.839; *P* = 0.002) were independently prognostic factors for DMFS (Table 3).

**Figure 2:**
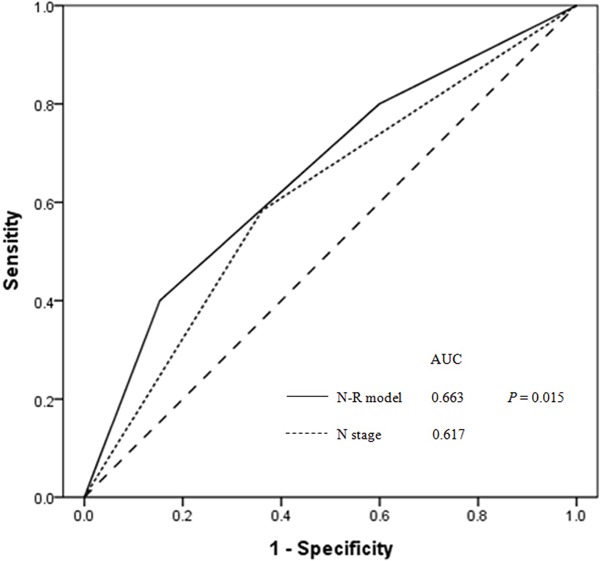
Receiver operator characteristic (ROC) curves for N-R model and N stage as predictors of distant metastasis for all NPC patients (n = 719) The area under the curve (AUC) was 0.663 for the N-R model (solid line) and 0.617 for clinical N stage (dotted line)(*P* = 0.015), indicating that the N-R model is a better predictor of distant metastasis.

As shown in the multivariate analysis, both CD4/CD8 ratio and clinical N stage were independent prognostic factors for DMFS. Based on CD4/CD8 ratio and clinical N stage, a N-R model was constructed as follows: (1) the low-risk group (early N stage and CD4/CD8 ratio ≥ 1.77) included 276 out of 719 (38.4%) patients; (2) the intermediate-risk group (advanced N stage or CD4/CD8 ratio < 1.77) included 318 out of 719 (44.2%) patients; and (3) the high-risk group (advanced N stage and CD4/CD8 ratio < 1.77) included 125 out of 719 (17.4%) patients. ROC curves were used to compare the prognostic validity of the N-R model and clinical N stage. In all patients, the AUC was 0.663 for the N-R model and 0.617 for clinical N stage (*P* = 0.015; Figure 2).

**Figure 3:**
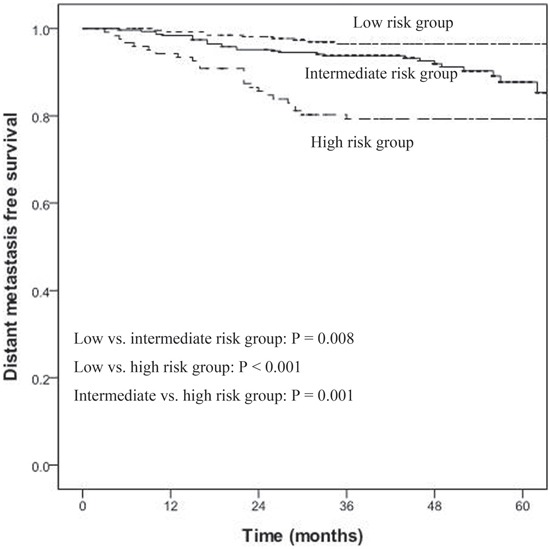
Distant metastasis-free survival (DMFS) for low-, intermediate-, and high-risk groups for all NPC patients (n = 719) Using pairwise comparisons, the DMFS of the intermediate- and high-risk groups were significantly poorer than the low-risk group (*P* = 0.008, *P* < 0.001; respectively) and the DMFS of intermediate-risk group was significantly better than the high-risk group (*P* = 0.001).

During the follow-up period, a total of 74 patients developed distant metastasis: 15 of 276 patients (5.4%) were in the low-risk group, 30 of 318 patients (9.4%) were in the intermediate-risk group and 29 of 125 patients (23.2%) were in the high-risk group (*P* < 0.001). The 5-year DMFS rates of the three groups were 95.4%, 85.3% and 79.3%, respectively (*P* < 0.001, Figure 3). Pairwise comparisons were performed among the three groups and showed that the DMFS of the intermediate- and high-risk groups were significantly poorer than the low-risk group (*P* = 0.008, *P* < 0.001; respectively). The DMFS of intermediate-risk group was significantly better than the high-risk group (*P* = 0.001) (Figure 3).

## DISCUSSION

In this study, we explored the correlation between the percentage of circulating immune subsets and clinical variables in NPC patients. Due to the fact that the lymphocytes plays a crucial role in immunity, especially anti-tumor immunity, the percentage of circulating lymphocyte subsets may be prognostic factors of NPC outcomes [15]. In this study, the percentage of CD19^+^ lymphocytes correlated negatively with TNM stage (r = −0.082, *P* = 0.028), which is consistent with the previous study [10]. This study also demonstrated several circulating lymphocyte subsets correlated with clinical T stage and N stage, suggesting that circulating lymphocyte subsets have a close relationship with tumor development and metastasis in NPC patients.

Previous reports have shown that the ratio of CD4/CD8 T cells reflects the immune system status as a surrogate marker of immunosenescence, and may independently predict all-cause mortality [12, 16]. Indeed, Shah *et al.* reported that the decreased CD4/CD8 ratio was significantly associated with the poorer prognosis of patients with cervical carcinoma [17]. In this study, we found a lower CD4/CD8 ratio is associated with poorer DMFS in patients with NPC.

The mechanisms by which lower CD4/CD8 ratio negatively influences DMFS in NPC patients may be explained by numerous confounding factors. Firstly, previous studies reported that the CD4/CD8 ratio was significantly lower in patients with higher tumor load and plasma concentration of Epstein-Barr virus DNA pre-treatment, indicating a higher risk of distant metastasis [12, 18]. Secondly, as CD4^+^ T cells play an anti-tumor role by enhancing cellular immune responses [19, 20], insufficient generation of CD4^+^ T cells could cause an inability of the host to reject the tumor [17]. Indeed, a decrease of CD4^+^ T cells has been associated with poor prognosis in advanced cancer [21, 22]. Similarly, the results from our study indicate that the CD4/CD8 ratio is an effective predictor of distant metastasis in NPC patients.

Previous studies have shown that the clinical outcome of NPC patients with the same TNM stage can vary widely, implying that the TNM staging system is suboptimal for predicting prognosis and metastasis [23]. As it is important to select patients for appropriate, individualized treatment by identifying patients at high-risk of distant metastasis, a better prognostic model is required. In this study, a simple and easily reproducible prognostic model (N-R model) was designed to predict the distant metastasis in NPC patients. This model was developed based on the N stage and CD4/CD8 ratio, both of which were significantly associated with distant metastasis in the multivariate analysis.

According to the N-R model, NPC patients were stratified depending on their N classification and CD4/CD8 ratio into three risk groups for distant metastasis: low-risk, intermediate-risk and high-risk groups. Based on ROC curves, we found that predicting metastasis in NPC patients could be improved by combining the N staging system with CD4/CD8 ratio. The 5-year DMFS rates of the low-risk, intermediate-risk and high-risk groups were 95.4%, 85.3% and 79.3%, respectively (*P* < 0.001).

There were several limitations in the current study. The nature of a retrospective analysis and that it was a single-institution experience inevitably brought selection bias and bias at the model-building stage. In addition, the validation set for the prognostic model were not created. A larger, prospective study that collaborates with other centers will be carried out to confirm the generalizability of this prognostic model.

In conclusion, our newly developed N-R model, that combines clinical N stage with CD4/CD8 ratio, may be better for predicting distant metastasis in NPC patients than using the TNM staging system alone. Such an improved prognostic ability would allow NPC patients with high risk of distant metastasis to receive more intensive treatment protocols, such as adjuvant chemotherapy or induced therapy.

## MATERIALS AND METHODS

### Patient characteristics

Between January 2007 and December 2012, 719 consecutive patients with newly diagnosed, non-metastatic and histologically proven NPC were treated with intensity-modulated radiation therapy (IMRT) in the Zhejiang Provincial Cancer Hospital. This study was approved by the institutional ethical review board of Zhejiang Provincial Cancer Hospital. Informed consent for the collection of medical information was obtained at each patient's first visit.

The pre-treatment work-up included complete history collection, physical examinations, hematology and biochemistry profiles, magnetic resonance imaging (MRI) of the nasopharynx and neck, chest radiography, a bone scan and an abdominal sonography. All patients were restaged according to the 7^th^ edition of the American Joint Commission on Cancer (AJCC) staging system [13]. The stage distribution was as follows: stage I, 14/719 (1.9%); stage II, 98/719 (13.6%); stage III, 381/719 (53.1%); stage IVa-b, 226/719 (31.4%).

### Treatment methods

All patients were immobilized in the supine position with a head, neck, and shoulder thermoplastic mask. We obtained two sets of images, i.e. with and without contrast, from the CT simulator for treatment planning. CT was performed after administering intravenous contrast medium, and we obtained 5 mm slices from the head to 1 cm below the sternoclavicular joint. The target volumes were delineated according to the International Commission on Radiation Units and Measurements reports 50 and 62. The clinical target volumes (CTV) were individually delineated based on the tumor invasion pattern as described previously [14]. The contoured images were transferred to a Pinnacle version 7.6 inverse IMRT planning system (Philips Medical Systems, Bothell, WA, USA). The prescribed radiation dose (i.e. the minimum dose received by the 95% of the planning target volume) was a total dose of 69-69.9 Gy in 30-33 fractions to the planning target volume (PTV) of the primary gross tumor volume (GTV), 67.5–69.9 Gy to the nodal GTV PTV, 60 Gy to the CTV-1 PTV (i.e. high-risk regions), and 54 Gy to the CTV-2 PTV (i.e. low-risk regions) and CTV-N (i.e. neck nodal regions). All patients were treated with one fraction daily over five days per week. All targets were treated simultaneously using the simultaneous integrated boost technique.

Overall, 21 patients were treated with radiotherapy alone, and 698 patients received concurrent chemoradiotherapy. Six hundred and forty-nine patients (90.3%) received neoadjuvant chemotherapy and 183 patients (25.5%) received adjuvant chemotherapy. Neoadjuvant or adjuvant chemotherapy consisted of cisplatin with 5-fluorouracil or taxanes every three weeks for two or three cycles. Concurrent chemotherapy consisted of cisplatin (80 mg/m2 intravenously in three daily doses) and was given every three weeks for two cycles.

### Determination of circulating lymphocyte subpopulations

The analysis of circulating lymphocyte subpopulations was performed on whole blood samples collected into heparinized tubes before the initiation of treatment. Heparinized blood (100 μL) was mixed with 20 μL of each of the following fluorescent mouse anti-human monoclonal antibodies according to the reagent instructions: CD3, CD4, CD8, CD19, CD25, CD44 and CD56 (Becton Dickinson, San Jose, CA). The mixtures were incubated for 30 minutes in the dark room, washed with phosphate buffered saline (PBS) (3 mL), and centrifuged at 1500 rpm for 5 minutes. The supernatant was discarded, and the pellet was resuspended with PBS (1 mL) for flow cytometric analysis. The percentage of fluorescent-positive cells was calculated with flow cytometry (Becton Dickinson). The experiments were repeated three times and the mean value was calculated for statistical analysis [10].

### Patient follow-up

Patients were regularly followed up after treatment every 3 months during the first 3 years, and every 6 months thereafter until death or their last follow-up appointment. The time of the final follow-up was December 2014, and the median follow-up duration was 48 months (ranging from 3 to 89 months). Physical examination and nasopharyngoscopy was performed routinely on each visit. Nasopharyngeal and neck MRI, chest X-ray, bone scan and abdominal sonogram were performed within three months after treatment or when clinical indications dictated.

### Statistical analysis

Survival duration was calculated from the first day after completing radiotherapy. Correlation between immune subsets and clinical variables was determined using the Spearman's rank correlation tests. The receiver operating characteristic (ROC) curve analysis was subjected to select the optimal cutoff points for distant metastasis with highest Youden's index (Youden's index = Sensitivity+Specificity-1). Kaplan–Meier analysis and log-rank test were used to compare the difference in survival rates. Multivariate analysis was performed with the Cox proportional hazards model to analyze factors related to prognosis. The criterion for statistical significance was set at α = 0.05; *P*-values were determined from two-sided tests. All statistical analyses were performed using SPSS v18.0 (SPSS, Chicago, IL, USA).
